# High CBX8 Expression Leads to Poor Prognosis in Laryngeal Squamous Cell Carcinoma by Inducing EMT by Activating the Wnt/β-Catenin Signaling Pathway

**DOI:** 10.3389/fonc.2022.881262

**Published:** 2022-06-23

**Authors:** Qingchao Meng, Lei Li, Liping Wang

**Affiliations:** ^1^ Department of Otolaryngology, Shengjing Hospital of China Medical University, Shenyang, China; ^2^ Department of Urology, ShengJing Hospital of China Medical University, Shenyang, China

**Keywords:** CBX8, LSCC, Wnt/β-catenin, EMT, TCGA

## Abstract

**Background:**

In this study, we detected the expression of chromobox protein homolog 8 (CBX8) in laryngeal squamous cell carcinoma (LSCC) and its influence on the occurrence and progression of LSCC.

**Methods:**

Pancancer analysis of CBX8 was analyzed by TCGA database and its expression in LSCC.The expression of CBX8 in 30 pairs of LSCC and adjacent tissues was analyzed by quantitative real-time PCR(qRT-PCR)and immunohistochemical assays, and its association with the prognosis and clinicopathological features of LSCC was further evaluated. A CBX8 knockdown model was constructed in AMC-HN-8 and Hep2 cell lines. The effects of CBX8 on LSCC cell proliferation, migration, invasion and apoptosis were detected by CCK8,EdU,wound healing, Transwell and flow cytometry assays. Levels of apoptosis-related protein, WNT/β-catenin signaling pathway and epithelial to mesenchymal transition (EMT) proteins, including Bax, Bcl2, β-catenin, DKK1, GSK3β, N-cadherin, E-cadherin and Snail1, in LSCC cells were detected by Western blotting.

**Results:**

CBX8 was overexpressed in LSCC. High expression of CBX8 in LSCC patients led to shorter overall survival and correlated with tumor stage and lymphatic metastasis. After CBX8 knockdown, the proliferation of AMC-HN-8 and Hep2 cells slowed, and the number of EdU-positive cells decreased. Wound healing slowed down, and the number of Transwell invading cells decreased. The percentage of apoptotic cells increased. The expression levels of Bcl2, β-catenin, N-cadherin and Snail11 proteins were significantly reduced in the CBX8 knockdown cells, while Bax, DKK1, GSK3β and E-cadherin significantly increased with their corresponding controls.

**Conclusion:**

CBX8 is highly expressed in LSCC and induces the EMT process by activating the Wnt/β-catenin signaling pathway to promote LSCC cell proliferation and migration and inhibit apoptosis, resulting in poor prognosis.

## Introduction

LSCC accounts for 85%-95% of all laryngeal cancers and is an important component of head and neck squamous cell carcinoma (HNSCC), which is also one of the most common tumors in the respiratory system and is highly invasive (1). There were 184,615 new laryngeal cancer cases, and 99,840 deaths were counted worldwide in 2020 according to a recent study from the International Agency for Research on Cancer (IARC) (2). The statistics in 2015 show that the number of new cases and deaths was 26,400 and 14,500 in China (3). Although the incidence of LSCC is relatively low compared with other types of cancer, it has been considered one of the leading causes of cancer-related deaths worldwide in recent years, especially in China (4). LSCC is treated with surgery, radiotherapy, chemotherapy or biological therapy, but the prognosis and survival did not improve significantly (5, 6). Therefore, searching for new therapeutic targets is crucial to LSCC patients.

Chromobox protein homolog 8 (CBX8), a core component of the polycomb group protein (PcG) PRC1 complex, can modify chromatin to inhibit the transcription of target genes. It plays a vital role in regulating cell proliferation, differentiation, senescence, death, tumorigenesis and metastasis (7, 8). The expression of CBX8 is closely related to the progression of a variety of cancers. Studies have shown that CBX8 is overexpressed in esophageal cancer, colorectal cancer, and breast cancer, resulting in a poor prognosis (9–12). Wnt/β-catenin signaling is a highly conserved signaling pathway that is overactivated in tongue squamous carcinoma, cervical cancer, lung cancer, gastric cancer and many other cancers (13–16). The imbalance of Wnt/β-catenin signaling in LSCC is one of the main reasons for the occurrence and progression of laryngeal cancer, and downstream proliferation signals are directly involved in tumor deterioration and metastasis (17). However, the expression of CBX8 in LSCC, its mechanism of action and its relationship with the Wnt/β-catenin signaling pathway have not been systematically elucidated. In our study, the expression of CBX8 in LSCC tissues and paired normal tissues was detected. By constructing LSCC cell lines with CBX8 knockdown, the impacts of CBX8 on cell proliferation, migration and apoptosis were observed, as well as changes in Wnt/β-catenin signaling and EMT-related proteins, to further explore the role of CBX8 in LSCC and its potential mechanism.

## Materials and Methods

### Integrative Analysis of TCGA and GESA

Pancancer analysis of CBX8 can be downloaded from cBioPortal (www.cbioportal.org). Transcriptome data (FPKM) of LSCC samples were downloaded from The Cancer Genome Atlas (TCGA) (https://portal.gdc.cancer.gov). GSE27020 was downloaded from the Gene Expression Omnibus (GEO) (https://www.ncbi.nlm.nih.gov/geo/). The CBX8 expression of tumor tissues in all LSCC patients was compared with that of paired normal tissues. The correlation between CBX8 and CTNNB1 in the TCGA COAD dataset was analyzed in the GEPIA database (http://gepia.cancer-pku.cn/). Gene set enrichment analysis (GSEA) was performed by GSEA 3.0 software.

### Clinical Specimens

A total of 30 patients with LSCC who underwent surgical resection in our hospital from August 2020 to March 2021 were enrolled and pathologically diagnosed with squamous cell carcinoma. The surgically removed cancer tissue samples were taken as the observation group, and the tissues 2 cm adjacent to the cancer were taken as the control group. All patients were primary cases and did not undergo preoperative radiotherapy or chemotherapy. Some specimens were placed in EP tubes filled with 4% paraformaldehyde and labeled for the preparation of paraffin sections. The remaining specimens were brought back to the laboratory and stored at -80°C. Another 10 pairs of LSCC and paracancer sections stored in the Department of Pathology of our hospital were collected. This study has been approved by the Ethics Committee of Shengjing Hospital affiliated to China Medical University (Ethics Number: 2017PS31K).

### Cell Culture

Two human LSCC cell lines (AMC-HN-8 and Hep2) were purchased from the Cell Bank of the Chinese Academy of Sciences (Shanghai, China). These cell lines were grown in DMEM (HyClone, Beijing, China) and cultured. T25 cell culture bottles were supplemented with 5 ml DMEM complete medium containing 1% penicillin–streptomycin and 10% fetal bovine serum (HAKA, China) and cultured in tin in a humidified chamber at 37°C with 5% CO2. When the cell density reached over 90%, it could be digested for subculture and frozen storage.

### Quantitative Real-Time PCR (qRT–PCR)

Thirty pairs of LSCC tissues and their paired adjacent normal tissues were collected during the operation and stored in a refrigerator at -80°C. RNA was extracted and reverse transcribed into cDNA according to Takara instructions. Using GAPDH as an internal reference, the relative expression of CBX8 mRNA in cancer and normal mucosal tissues was detected. CBX8 forward primer 5 ‘-TTATTTCTGGGTGGGATGTGG-3’, reverse primer 5’-GGGAGGAAGGAGGGATGAA-3’; GAPDH forward primer 5’-TTATTTCTGGGTGGGATGTGG-3’, reverse primer 5’-GGGAGGAAGGAGGGGAA-3’. Fluorescence quantitative signals were analyzed by the 2^-ΔΔCt method.

### Immunohistochemistry

Tissue wax blocks were cut to a thickness of 3.5 µm and hydrated in different concentrations of xylene and ethanol. Then, the antigen was repaired by microwave heating in citrate for 8 min. Next, an SP Kit (SP-9000; Zsgb-bio, Beijing, China) was used according to the manufacturer’s instructions. First, endogenous peroxidase blockers were incubated for 30 minutes, phosphate-buffered saline (PBS) was added 3 times, and 10 minutes later, normal goat serum was added for 30 minutes. All the above operations were carried out at room temperature. Subsequently, the blocked tissue sections were incubated with an anti-CBX8 rabbit polyclonal antibody (1:200, NOVUS Biologicals USA) overnight at 4°C. Binding antibodies were detected by the biotin-streptavidin-peroxidase method and visualized by DAB as a chromogenic agent. Slices were stained with hematoxylin and sealed with neutral resin after dehydration. PBS should be used as a negative control in place of CBX8 antibody.

### Cell Transfection

CBX8-siRNA and negative control siRNA were designed by Gima Genomics. AMC-HN-8 and Hep2 cells were inoculated on 6-well plates and transfected 5µl siRNA and 5µl Lipofectamine 3000 (Invitrogen, Carlsbad, CA, USA) to each well when the degree of fusion reached 50%. After culturing for 48 h, the cell RNA and protien samples obtained were tested for siRNA knockdown efficiency by qRT–PCR and Western Blot. The siRNA with the highest knockdown efficiency was selected for the following functional experiments. The siRNA sequences sense:5’-GAAGUACAGCACAUGGGAATT-3’, antisense: 5’-UUCCCAUGUGCUGUACUUCTT-3’. Next, *in vitro* experiments were divided into control, si-Negtive Control (si-NC) and si-CBX8 groups.

### Cell Counting Kit-8 Assay

Cell Counting Kit-8 (CCK-8) was used to detect cell proliferation in each group. Twenty-four hours after transfection, 100 µL AMC-HN-8 and Hep2 cell suspensions at a density of 3x10^4^ cells/mL were added to each well of the 96-well plate. Three secondary wells were repeated in each group. Then, 100 µL of complete culture medium containing 10% CCK8 reagent was added to each well after the cells returned to normal form and incubated at 37°C for 2 h in the dark. The absorbance at a wavelength of 450 nm was measured with a microplate reader (BioTek Instruments, Winooski, USA). The above operations were repeated at 24 h, 48 h and 72 h.

### 5-Ethynyl-2’-Deoxyuridine (EdU) Assay

In addition, an EdU proliferation assay can also be used to detect cell proliferation. Twenty-four hours after transfection with 6-well plates, a mixture of 1% EdU (Beyotime, Shanghai, China) and DMEM was added to each well and cultured in a cell incubator for an extra 2 h. Next, 4% formaldehyde fixation, fluorescence staining and other specific procedures were carried out according to the reagent instructions. Five fields were randomly selected under a fluorescence microscope (Olympus, Tokyo, Japan) to analyze the proportion of EdU-positive cells.

### Wound Healing Assay

We observed cell migration by wound healing tests. The cells were plated in 6-well plates, and when the degree of cell fusion after transfection reached more than 90%, a straight wound was made with a 100 µL pipette tip under the ruler mark. PBS was gently cleaned to remove suspended cells 3 times and photographed under a microscope. Culture was continued for 24 hours after serum-free medium was added to each well. PBS was also used for cleaning, and wound healing was observed by photographing again.

### Transwell Invasion Assay

The day before the experiment, 100µL diluted Matrigel was added to the upper chamber of Transwell chamber and placed in an incubator overnight. Twenty-four hours after transfection, 200 µL serum-free cell suspension containing 3x10^4^ cells was added to the upper chamber, and 500 µL medium containing 10% serum was added to the lower chamber and then incubated at 37°C for 24 h. Next, the chambers were removed from the 24-well plate, and the cells that did not penetrate the upper chamber were gently wiped with cotton swabs after fixation with 4% paraformaldehyde and staining with 0.1% crystal violet. Images were taken by light microscopy, and cells in each experimental group were counted by ImageJ software.

### Flow Cytometry Apoptosis Assay

Twenty-four hours after transfection, LSCC cells were digested by trypsin and washed with PBS 3 times. The cells were suspended by flow tube with 1X Binding Buffer density of 1×10^6^ cells/ML.100 µL cell mixed suspension was added to each tube, followed by 5 µL Annexin V-FITC and 5 µL PI staining successively. After 15 min of reaction, another 400 µL of 1X binding buffer was added to stop the reaction. Finally, detecting within 1 h. The percentage of apoptotic cells in each group was assessed by calculating the sum of Annexin V +PI+ and Annexin V +PI- cells.

### Transmission Electron Microscopy(TEM)

Twenty-four hours after transfection, the cells were digested by trypsin and washed 3 times with PBS. The cells were fixed with 2.5% glutaraldehyde and 1% osmium solution at 4°C for 2 h and then rinsed with sodium dimethoarate 3 times for 5 min each. The cells were dehydrated with ethanol and acetone of different concentration gradients, soaked with a mixed solution of epoxy resin and acetone, and finally embedded, sectioned and photographed under a microscope.

### Western Blotting

Forty-eight hours after transfection, the cells were centrifuged at 14000×rpm and 4°C for 15 min for total protein extraction and quantified by the BCA method. Subsequently, 30μg of each protein sample was subjected to SDS-PAGE electrophoresis, and then the samples were transferred to PVDF membranes and blocked with 5% skim milk for 2 h. After blocking, the corresponding primary antibodies CBX8(1:10000), Bcl2(1:1000), Bax(1:1000), β-catenin(1:1000), DKK1(1:1000), GSK3β(1:1000), N-cadherin(1:1000), E-cadherin(1:1000), Snail1(1:1000), GAPDH(1:5000)were incubated with the membrane overnight at 4°C. On the second day, after removing the primary antibodies and washing the membranes with Tween-20 tris buffered saline (TBST), the corresponding secondary antibody (1:5000) was incubated for 2 h. Then, the cells were washed with TBST again, and ECL hypersensitive chromogenic solution was added for development and photography. Imaje J software was used to process all the transfer strips, and the gray values of each strip were obtained for statistical analysis.

### Statistical Analysis

SPSS 22.0 and GraphPad Prism 9 software were used for experimental data analysis and statistical graph drawing. The data are presented as the mean values ± SEMs. Independent sample *T* tests were used for comparisons between the two samples, and paired samples were used for paired *t* tests. The correlation between CBX8 expression and clinicopathological features was estimated by Pearson’s *X* ^2^ test. The Kaplan–Meier method was used to draw the survival curve, and the log-rank method was used to test the difference. Cox regression analysis was used to analyze univariate and multivariate factors affecting the prognosis of patients. *P* < 0. 05 was statistically significant.

## Results

### CBX8 Is Overexpressed in Various Cancers and LSCC Tissues

**Figure 1:**
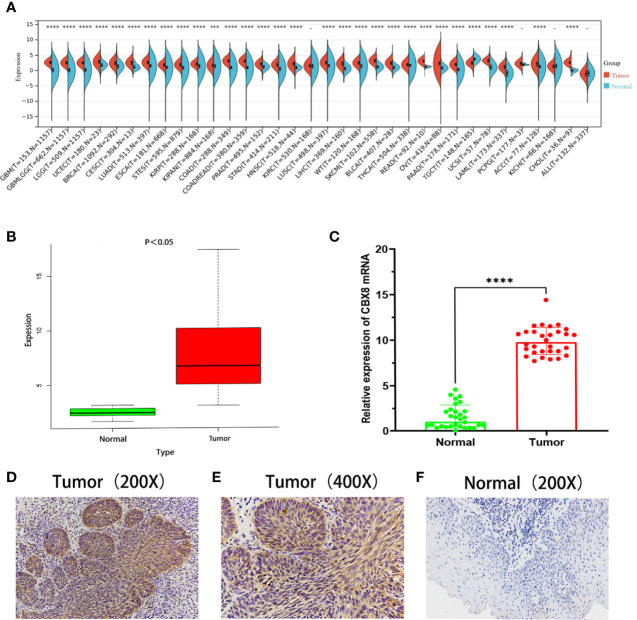
CBX8 was highly expressed in LSCC tissues. **(A)** Expression of CBX8 mRNA in different cancer types according to TCGA and GTEx data. **(B)** Expression of CBX8 in laryngeal squamous cell carcinoma in TCGA database. **(C)** Real-time quantitative PCR was used to detect the relative expression of CBX8 mRNA in laryngeal squamous cell carcinoma and paired normal tissues. **(D–F)** IHC detected the expression of CBX8 protein in laryngeal carcinoma and paired normal tissues. (*P < 0.05, ***P < 0.001, ****P < 0.0001).

The expression of the CBX8 gene in various common tumors and LSCC was compared and analyzed in the TCGA database. The CBX8 gene is highly expressed in 30 types of malignant tumors, including HNSCC, and expressed at low levels in 4 types of tumors (**Figure 1A**). According to the transcriptomic data of TCGA, the expression of CBX8 mRNA in LSCC was significantly higher than that in para-cancer tissues by using the “limma” package (P<0.001)(**Figure 1B**). Meanwhile, cancer tissues of 30 LSCC patients who underwent total laryngeal surgery in Shengjing Hospital affiliated with China Medical University and their paired para-cancer tissues were collected to detect the expression of CBX8 mRNA by qRT–PCR. Consequently, the results obtained were consistent with TCGA database analysis, and the difference was significant (P<0.001) (**Figure 1C**). Furthermore, IHC revealed that CBX8 is mainly expressed in the tumor nucleus and cytoplasm, and its expression in LSCC (**Figures 1D, E**) was significantly higher than that in paracancer tissues (**Figure 1F**), positive rate in cancer tissues and paracancer tissues were respectively 67.5%(27/40)and 22.5% (9/40)(**Table 1**). These data suggest that CBX8 was overexpressed in LSCC.

**Table 1:** Expression of CBX8 protein in LSCC.

Group	n	Positive	Negative	Positive rate (%)	*X^2^*	*P*
LSCC tissue	40	27	13	67.5	16.364	0.000
Normal paracancer tissue	40	9	31	22.5		

### High Expression of CBX8 Leads to Poor Prognosis in Patients With LSCC

**Figure 2:**
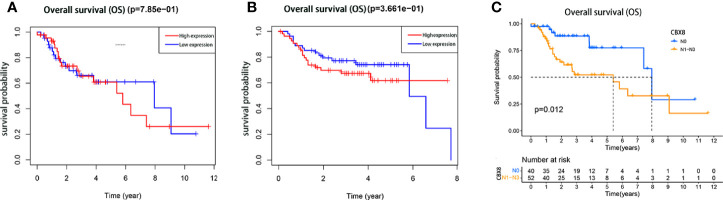
The relationship between the expression of CBX8 protein and the prognosis of patients with LSCC. Kaplan–Meier survival curve comparison of high and low expression of CBX8. **(A)** Kaplan–Meier analysis of TCGA samples. **(B)** The Kaplan–Meier analysis of the GEO (GSE27020)samples. **(C)** The Kaplan–Meier analysis of the TCGA samples with or without lymph node metastasis.

We divided the expression level of CBX8 in 111 TCGA tumor samples into high expression and low expression groups according to the median(6.690479164). The overall survival rate of CBX8 obtained by the “survival” package showed that the survival time of patients with high CBX8 expression was shorter than that of patients with low CBX8 expression (**Figure 2A**). Next, we used the GEO database for verification, and the conclusion was consistent with the previous analysis (**Figure 2B**). Meanwhile, a total of 92 patients with definite lymph node typing were screened from TCGA database, our bioassay results showed that its expression level was also associated with lymph node metastasis, and survival rates were higher in early-stage patients than in advanced-stage patients (**Figure 2C**). IHC came to the same conclusion, the positive expression rate was 92.31% (12/13) in patients with lymph node metastasis and 55.56% (15/27) in patients without lymph node metastasis. The expression level of CBX8 protein in LSCC tissues was also correlated with the clinical stage of patients. The positive expression rates of STAGE I & II and III & IV patients were 18.18% (2/11), 86.21% (25/29), respectively, with statistically significant differences. At the same time, there was no correlation between CBX8 protein expression level and sex, age, smoking, drinking, site of disease or differentiation degree of patients (**Table 2**).

**Table 2:** The relationship between CBX8 protein expression and clinicopathological features.

Clinical features		n (40)	CBX8		X*^2^*	P-value
			positive (n)	negative (n)		
Gender	Male	34	24	10	0.985	0.321
	Female	6	3	3		
Age	≥60	27	18	9	0.026	0.871
	<60	13	9	4		
Smoking	Yes No	38 2	26 1	12 1	0.294	0.588
Drinking	Yes No	30 10	19 8	11 2	0.950	0.330
Location	Supraglott	14	11	3	2.664	0.264
	Vocal	24	14	10		
	Infraglott	2	2	0		
The degree of differentiation	Low	7	2	5	5.875	0.053
	Medium	20	15	5		
	High	13	10	3		
T stage	l/ll	11	2	9	16.822	0.000
	III/IV	29	25	4		
N stage	N0	27	15	12	5.403	0.020
	N1/N2	13	12	1		
M stage	M0	38	26	12	0.294	0.588
	M1	2	1	1		

### Promotive Effect of CBX8 on the Proliferative, Migrative and Invasive Ability of LSCC

**Figure 3:**
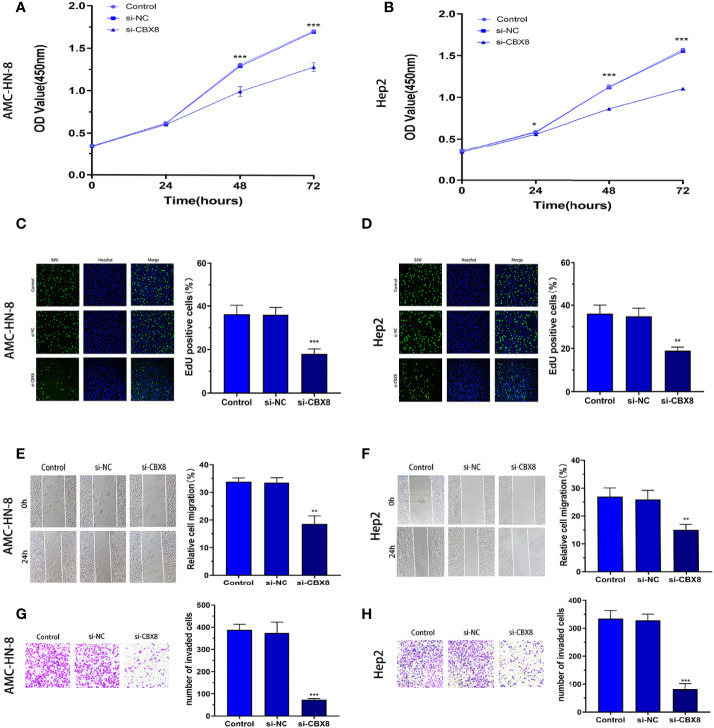
Knockdown of CBX8 inhibited LSCC cell proliferation, migration and invasion. **(A, B)** A CCK-8 assay was used to detect the effect of CBX8 on the proliferation of LSCC cells. **(C, D)** The effect of CBX8 on the proliferation of LSCC was detected by EdU assay. **(E, F)** The effect of CBX8 on the migration of LSCC cells was detected by wound healing assay. **(G, H)** Transwell assays were used to detect the effect of CBX8 on the invasion of LSCC cells (crystal violet staining ×200). Each assay was repeated at least three times. Data from one representative experiment is presented as Mean ± SEM (**P < 0.01, ***P < 0.001).

To observe the effect of CBX8 on the biological behavior of LSCC cells, we synthesized CBX8 siRNA and transfected AMC-HN-8 and Hep2 cells with the highest knockdown efficiency. Finally, we observed that after CBX8 knockdown, the expression of CBX8 mRNA in AMC-HN-8 and Hep2 cells was significantly decreased. The CCK-8 results showed that the proliferation ability of AMC-HN-8 and Hep2 cells was significantly weakened after CBX8 knockdown (**Figures 3A, B**). EdU experiments further verified the above findings, and the number of EdU-positive cells in the CBX8-siRNA group was significantly reduced, which also indicated that cell proliferation was weakened (**Figures 3C, D**). In the wound healing test, the migration distance of the CBX8-siRNA group to the wound was significantly shorter (**Figures 3E, F**). Transwell invasion assay showed that the number of cell invasion was significantly reduced after CBX8 knockdown (**Figures 3G, H**).

### Knockdown of CBX8 Can Promote Apoptosis in LSCC Cells

**Figure 4:**
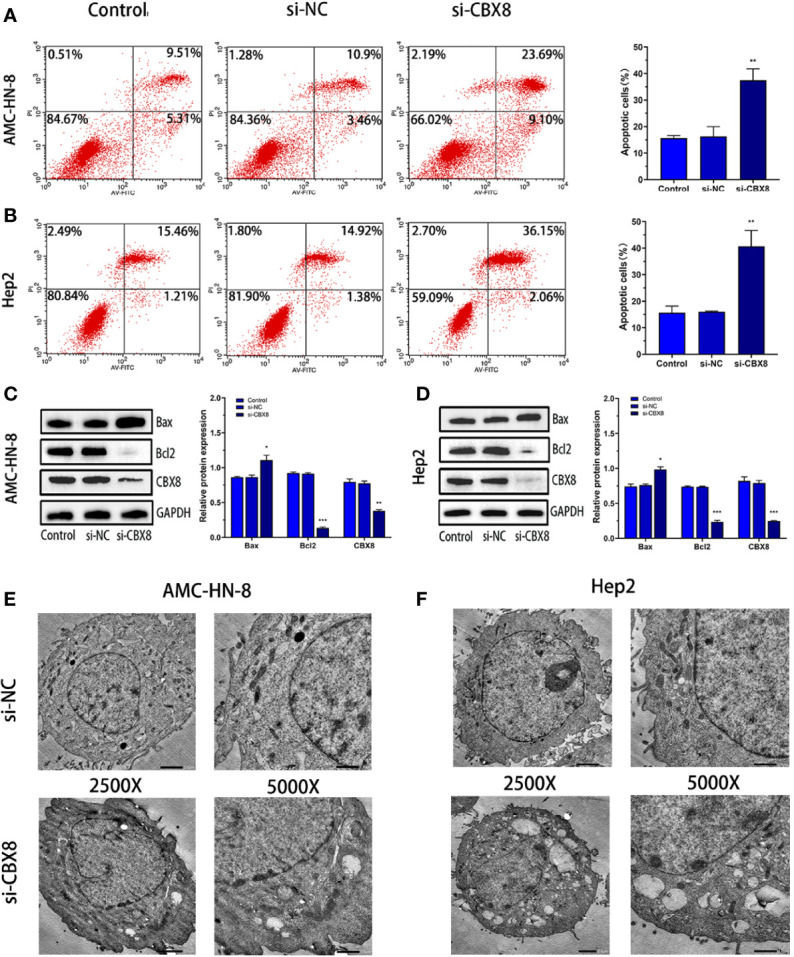
Knockdown of CBX8 can promote apoptosis and autophagy in LSCC cells. **(A**, **B)** After knockdown of CBX8, cell apoptosis in each group was detected by flow cytometry. **(C, D)** Western blotting was used to detect the protein expression of CBX8, Bax and Bcl2 in AMC-HN-8 and Hep2 cells.**(E**, **F)** Morphological changes in CBX8 cells were observed by transmission electron microscopy.Each assay was repeated at least three times. Data from one representative experiment is presented as Mean ± SEM (*P < 0.05, **P < 0.01, ***P < 0.001).

**A** CBX8 knockdown model was constructed by siRNA transfection, and apoptosis was detected by flow cytometry (Annexin V/PI method). The results showed that the apoptosis rate of the two LSCC cell lines increased significantly in the si-CBX8 groups (**Figures 4A, B**). Meanwhile, total protein of each group was extracted, and Western blotting confirmed that the expression of the apoptotic protein Bax increased and Bcl2 decreased with the decline of CBX8 (**Figures 4C, D**). Furthermore, transmission electron microscopy showed that after the knockdown of CBX8 expression, the nuclei of AMC-HN-8 and Hep2 cells were deformed, mitochondria were obviously damaged, normal morphology disappeared, and large vacuolar structures were formed (**Figures 4E, F**).

### Knockdown CBX8 Regulates Wnt/β-Catenin Signaling Pathway

**Figure 5:**
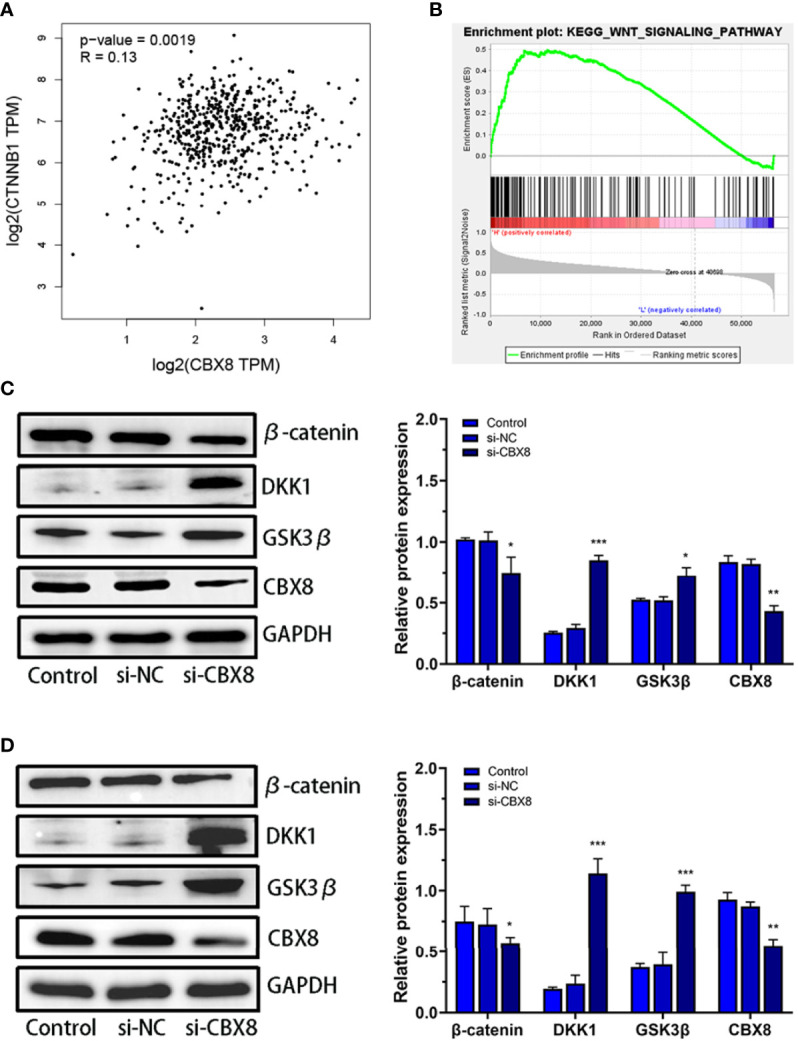
Effect of CBX8 knockdown on the Wnt/β-catenin signaling pathway. **(A)** Correlation analysis between CBX8 and CTNNB1 in the TCGA CO-AD dataset. **(B)** GSEA showed that CBX8 was enriched in the Wnt/β-catenin signaling pathway in LSCC. **(C, D)** The expression levels of β-catenin, DKK1 and GSK3β in AMC-HN-8 and Hep2 cells were detected by Western blotting and semiquantitative analysis. Each assay was repeated at least three times. Data from one representative experiment is presented as Mean ± SEM (*P < 0.05, **P < 0.01, ***P < 0.001).

The CTNNB1 gene encodes the β-catenin protein, which is an important regulatory protein of the Wnt/β-catenin signaling pathway (18). Correlation analysis between CBX8 and CTNNB1 in the TCGA CO-AD dataset showed that CBX8 was positively correlated with CTNNB1 gene expression (**Figure 5A**). To elucidate the mechanism by which CBX8 promotes cell proliferation, we searched for pathways through which CBX8 might be involved *via* KEGG enrichment analysis, and the results showed that CBX8 was associated with the Wnt signaling pathway (**Figure 5B**). Based on this, we detected changes in Wnt/β-catenin signaling pathway-related proteins in CBX8 knockdown cells. The results showed that the expression of β-catenin protein in AMC-HN-8 and Hep2 cells decreased, while the expression of DKK1 and GSK3β increased after transfection (**Figures 5C, D**). These results suggest that CBX8 may positively regulate the Wnt/β-catenin signaling pathway to promote LSCC cell proliferation and migration and inhibit apoptosis.

### Knockdown CBX8 Regulates EMT-Related Protein Expression

**Figure 6:**
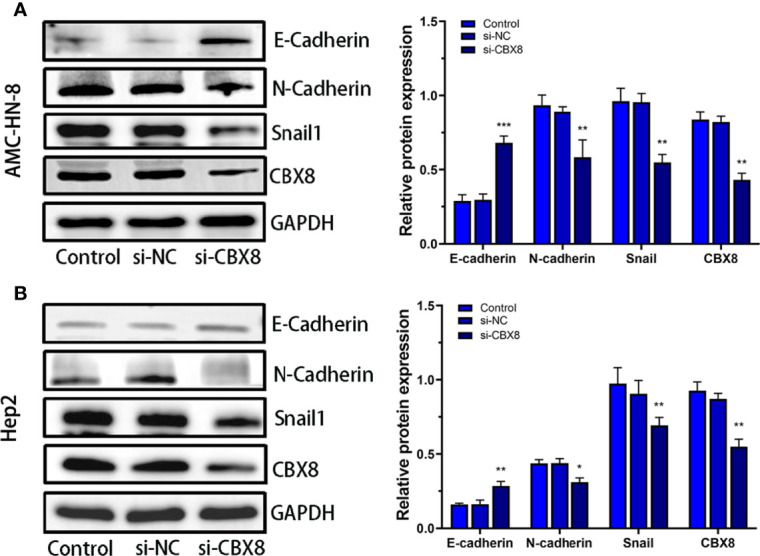
Effect of CBX8 knockdown on EMT-related proteins. Western blotting was used to detect the expression of E-cadherin, N-cadherin and Snail1 in AMC-HN-8 cells **(A)** and Hep2 cells **(B)** and semiquantitative analysis.Each assay was repeated at least three times. Data from one representative experiment is presented as Mean ± SEM (*P < 0.05, **P < 0.01, ***P < 0.001).

Relevant studies have confirmed that CBX8 can induce tumor cell proliferation and migration by regulating EMT (19). We detected changes in the EMT-related proteins Snail1, E-catherine and N-cadherin after transfection. Western blotting analysis clearly showed that E-Catherine (epithelial marker) significantly increased while N-cadherin and Snail1 protein levels decreased in CBX8-knockdown cellss (**Figures 6A, B**).

## Discussion

The incidence of LSCC is the second highest among all types of HNSCC, and the overall survival rate is still low (20, 21). Therefore, it is of great significance to explore the molecular mechanism of LSCC disease progression. The PcG protein family is a family of proteins that inhibit gene expression epigenetics through chromatin remodeling and histone modification (22) and is involved in stem cell differentiation and tumorigenesis and metastasis (23, 24). CBX8 belongs to the PcG protein family and can jointly induce and maintain the inhibition state of the gene with PRC2 by combining with RING1a/B, BMI1 and other protein molecules to form PRC1 (8). Recent studies have shown that CBX8 interacts with chromatin PTEN to regulate mitosis (25). High expression of CBX8 in HCC enhances proliferation and migration by activating the AKT/β-catenin signaling pathway (26), while in glioblastoma cells, breast cancer cells and lung cancer cells, CBX8 can strengthen proliferation and invasion capacity by activating the WNK2/MMP2 signaling pathway, ultimately leading to metastasis (12). In this study, through the analysis of TCGA database, we can clearly see that CBX8 is highly expressed in a large number of common malignant tumors, including HNSCC, so we speculate that CBX8 may also be involved in the progression of LSCC. Subsequently, through R language analysis in TCGA database, CBX8 mRNA was highly expressed in LSCC tissues. This result was confirmed in our clinical patient tissue collection. TAGA and GEO database analysis showed that the survival rate of patients with high expression of CBX8 tumors was lower.TCGA database analysis further showed that the survival rate of patients with lymph node metastasis was shorter than that of patients without lymph node metastasis. In addition, Zeng et al. (27) also confirmed that the expression of CBX8 was inversely proportional to the survival rate of patients with bladder cancer and was highly expressed in patients with stage III and IV advanced tumors and lymph node metastasis, which was consistent with the results obtained in our analysis of CBX8 and clinicopathological features.These results suggested that high expression of CBX8 leads to poor prognosis in LSCC patients. By constructing a CBX8 knockdown model in LSCC cell lines, cell phenotype tests suggested that CBX8 silencing can inhibit cell proliferation, migration,invasion and promote apoptosis. Transmission electron microscopy showed that the nucleus was deformed, mitochondria disappeared and autophagosomes formed. Many studies have proven that autophagy may promote apoptosis (28), further demonstrating that CBX8 can inhibit cell apoptosis. These studies suggested that CBX8 may be inextricably linked to the progression of LSCC.

The occurrence and progression of LSCC are regulated by a variety of signaling pathways. Abnormally activated Wnt/β-catenin signaling pathway regulates the occurrence and development of a variety of tumors, promoting progression, metastasis, and poor prognosis (29, 30). For example, Yu et al. (31) identified a novel UBE2T inhibitor, M435-1279, and showed that M435-1279 inhibits overactivation of the Wnt/β-catenin signaling pathway by blocking UBE2T-mediated RACK1 degradation, thereby inhibiting gastric cancer progression and reducing cytotoxicity. Abnormal regulatory transcription factor β-catenin is the key to initiating the Wnt/β-catenin pathway, which can lead to early carcinogenesis (32). In addition, Wnt ligand inhibits ubiquitination and degradation of β-catenin, which is a downstream target gene of the costimulator activation signal of TCF/LEF family transcription factors (33). DKK1 and GSK3β are important components involved in the Wnt/β-catenin signaling pathway. High expression of DKK1 can reduce intracellular β-catenin expression and promote apoptosis of WB-F344 cells (34). Active GSK3β binds to CK1 (Casein Kinase 1), APC (Adenomatous Polyposis Coli), AXIN1 and other members of the destruction complex to inhibit tumor development by phosphorylation and subsequent degradation of the oncogenic β-catenin protein (35). In this study, Western blotting data showed that the expression of β-catenin was also decreased after the expression of CBX8 was downregulated, while the expression of DKK1 and GSK3β was increased, suggesting that the high expression of CBX8 in LSCC may positively regulate the Wnt/β-catenin signaling pathway.

EMT is a reversible developmental process in which cancer cells reversibly transition from an epithelial phenotype of apex-basal polarity and cell adhesion to a fusiform and more active mesenchymal state, enabling cell migration and invasion (36, 37); the result is multiple tumor metastases and poor prognosis (38). EMT transformation in LSCC has been reported. For example, Sun et al. (39) found that DHA could inhibit EMT induced by IL-6 in LSCC cells through the miR-130B-3p/STAT3/β-catenin pathway, and the interaction between EMT and angiogenesis can improve the invasivity of LSCC (40). We concluded that the expression of E-cadherin increased after CBX8 knockdown, while the expression of N-cadherin and Snail1 decreased, suggesting that CBX8 may participate in the EMT process in LSCC. Many studies have proven that activation of the Wnt/β-catenin signaling pathway can lead to deterioration by inducing EMT in many different types of cancer (41). In other studies, reduced levels of E-cadherin and its complexes were inversely associated with tumor histological grade and were directly associated with intrahepatic metastasis and liver capsule invasion (42). Other studies also found that Galectin-3 could directly increase the expression of N-cadherin by promoting the accumulation of β-catenin in the nucleus, which activated the transcription of TCF/LEF-responsive genes (43). In ovarian cancer tissues and cell lines, GOLPH3 (high phosphoprotein 3) is a gene that encodes an oncoprotein, and Snail1 and other EMT transcription factors have been shown to regulate its expression by activating the Wnt/β-catenin signaling pathway (44). The occurrence of EMT in LSCC is also associated with the signal. Li et al. (45) found that CDK8 was involved in the progression of EMT through β-catenin of Wnt signaling. Therefore, combined with the above results, it can be speculated that high expression of CBX8 may induce the occurrence of LSCC EMT by regulating the Wnt/β-catenin signaling pathway.

Our study analyzed the expression of CBX8 in LSCC and adjacent normal mucosal tissues and its relationship with the Wnt/β-catenin signaling pathway and EMT. CBX8 may regulate the expression of β-catenin, DKK1, GSK3β and EMT-related proteins by activating the Wnt/β-catenin signaling pathway in LSCC, thus enhancing LSCC cell proliferation, migration and invasion, inhibiting apoptosis, and ultimately leading to poor prognosis of patients. Overall, CBX8 may be intimately involved in the diagnosis and treatment of LSCC, but the specific action and mechanism need to be further studied.

## Data Availability Statement

The original contributions presented in the study are included in the article/supplementary material. Further inquiries can be directed to the corresponding author.

## Ethics Statement

The studies involving human participants were reviewed and approved by the ethics committee of Shengjing Hospital Affiliated to China Medical University (ethics No.: 2017ps31k). Written informed consent for participation was not required for this study in accordance with the national legislation and the institutional requirements. Written informed consent was not obtained from the individual(s) for the publication of any potentially identifiable images or data included in this article.

## Author Contributions

QM conceived structure, authored, or reviewed drafts of the paper, approved the final draft, writing - Review and Editing, prepared figures and/or tables, and approved the final draft. LL: assisting to search related literature. LW authored or reviewed drafts of the paper, and approved the final draft.

## Conflict of Interest

The authors declare that the research was conducted in the absence of any commercial or financial relationships that could be construed as a potential conflict of interest.

## Publisher’s Note

All claims expressed in this article are solely those of the authors and do not necessarily represent those of their affiliated organizations, or those of the publisher, the editors and the reviewers. Any product that may be evaluated in this article, or claim that may be made by its manufacturer, is not guaranteed or endorsed by the publisher.
